# Supplementary material for: Association between gut microbiota and allergic rhinitis: a systematic review and meta-analysis
Source: PeerJ. 2025 May 26;13:e19441. doi: 10.7717/peerj.19441 (PMC12121621; doi:10.7717/peerj.19441)
Supplement: Supplemental Information 1 [file peerj-13-19441-s001.docx]

Supplementary Table 1. Search terms for each electronic database

| Database | Retrieval formula |
| --- | --- |
| Pubmed | (((Rhinitis, Allergic[MeSH Terms]) OR (Rhinitis, Allergic, Seasonal[MeSH Terms])) OR (Allergic Rhinitides [Title/Abstract]) OR (Rhinitides, Allergic [Title/Abstract]) OR (Allergic Rhinitis[Title/Abstract]) OR (Seasonal Allergic Rhinitis[Title/Abstract]) OR (Allergic Rhinitides, Seasonal[Title/Abstract]) OR (Allergic Rhinitis, Seasonal[Title/Abstract]) OR (Rhinitides, Seasonal Allergic[Title/Abstract]) OR (Rhinitis, Seasonal Allergic[Title/Abstract]) OR (Seasonal Allergic Rhinitides[Title/Abstract]) OR (Pollen Allergy[Title/Abstract]) OR (Allergies, Pollen[Title/Abstract]) OR (Allergy, Pollen[Title/Abstract]) OR (Pollen Allergies[Title/Abstract]) OR (Pollinosis[Title/Abstract]) OR (Pollinoses[Title/Abstract]) OR (Hay Fever[Title/Abstract]) OR (Fever, Hay[Title/Abstract]) OR (Hayfever[Title/Abstract]) OR (nasal allergy[Title/Abstract]) OR (rhinallergosis[Title/Abstract])) AND ((Gastrointestinal Microbiome[MeSH Terms]) OR (Gastrointestinal Microbiomes[Title/Abstract]) OR (Microbiome, Gastrointestinal[Title/Abstract]) OR (Gut Microbiome[Title/Abstract]) OR (Gut Microbiomes[Title/Abstract]) OR (Microbiome, Gut[Title/Abstract]) OR (Gut Microflora[Title/Abstract]) OR (Microflora, Gut[Title/Abstract]) OR (Gut Microbiota[Title/Abstract]) OR (Gut Microbiotas[Title/Abstract]) OR (Microbiota, Gut[Title/Abstract]) OR (Gastrointestinal Flora[Title/Abstract]) OR (Flora, Gastrointestinal[Title/Abstract]) OR (Gut Flora[Title/Abstract]) OR (Flora, Gut[Title/Abstract]) OR (Gastrointestinal Microbiota[Title/Abstract]) OR (Gastrointestinal Microbiotas[Title/Abstract]) OR (Microbiota, Gastrointestinal[Title/Abstract]) OR (Gastrointestinal Microbial Community[Title/Abstract]) OR (Gastrointestinal Microbial Communities[Title/Abstract]) OR (Microbial Community, Gastrointestinal[Title/Abstract]) OR (Gastrointestinal Microflora[Title/Abstract]) OR (Microflora, Gastrointestinal[Title/Abstract]) OR (Gastric Microbiome[Title/Abstract]) OR (Gastric Microbiomes[Title/Abstract]) OR (Microbiome, Gastric[Title/Abstract]) OR (Intestinal Microbiome[Title/Abstract]) OR (Intestinal Microbiomes[Title/Abstract]) OR (Microbiome, Intestinal[Title/Abstract]) OR (Intestinal Microbiota[Title/Abstract]) OR (Intestinal Microbiotas[Title/Abstract]) OR (Microbiota, Intestinal[Title/Abstract]) OR (Intestinal Microflora[Title/Abstract]) OR (Microflora, Intestinal[Title/Abstract]) OR (Intestinal Flora[Title/Abstract]) OR (Flora, Intestinal[Title/Abstract]) OR (Enteric Bacteria[Title/Abstract]) OR (Bacteria, Enteric[Title/Abstract])) |
| Ovid databases(Ovid MEDLINE) | (Rhinitis, Allergic OR Rhinitis, Allergic, Seasonal OR Allergic Rhinitides OR Rhinitides, Allergic OR Allergic Rhinitis OR Seasonal Allergic Rhinitis OR Allergic Rhinitides, Seasonal OR Allergic Rhinitis, Seasonal OR Rhinitides, Seasonal Allergic OR Rhinitis, Seasonal Allergic OR Seasonal Allergic Rhinitides OR Pollen Allergy OR Allergies, Pollen OR Allergy, Pollen OR Pollen Allergies OR Pollinosis OR Pollinoses OR Hay Fever OR Fever, Hay OR Hayfever OR nasal allergy OR rhinallergosis) AND (Gastrointestinal Microbiome OR Gastrointestinal Microbiomes OR Microbiome, Gastrointestinal OR Gut Microbiome OR Gut Microbiomes OR Microbiome, Gut OR Gut Microflora OR Microflora, Gut OR Gut Microbiota OR Gut Microbiotas OR Microbiota, Gut OR Gastrointestinal Flora OR Flora, Gastrointestinal OR Gut Flora OR Flora, Gut OR Gastrointestinal Microbiota OR Gastrointestinal Microbiotas OR Microbiota, Gastrointestinal OR Gastrointestinal Microbial Community OR Gastrointestinal Microbial Communities OR Microbial Community, OR Gastrointestinal OR Gastrointestinal Microflora OR Microflora, Gastrointestinal OR Gastric Microbiome OR Gastric Microbiomes OR Microbiome, Gastric OR Intestinal Microbiome OR Intestinal Microbiomes OR Microbiome, Intestinal OR Intestinal Microbiota OR Intestinal Microbiotas OR Microbiota, Intestinal OR Intestinal Microflora OR Microflora, Intestinal OR Intestinal Flora OR Flora, Intestinal OR Enteric Bacteria OR Bacteria, Enteric) |
| Cochrane library | #1 MeSH descriptor: [Rhinitis, Allergic] explode all trees  #2 (Rhinitis, Allergic OR Allergic Rhinitides OR Rhinitides, Allergic OR Allergic Rhinitis):ti,ab,kw (Word variations have been searched)  #3 MeSH descriptor: [Rhinitis, Allergic, Seasonal] explode all trees  #4 (Rhinitis, Allergic, Seasonal OR Seasonal Allergic Rhinitis OR Allergic Rhinitides, Seasonal OR Allergic Rhinitis, Seasonal OR Rhinitides, Seasonal Allergic OR Rhinitis, Seasonal Allergic OR Seasonal Allergic Rhinitides OR Pollen Allergy OR Allergies, Pollen OR Allergy, Pollen OR Pollen Allergies OR Pollinosis OR Pollinoses OR Hay Fever OR Fever, Hay OR Hayfever):ti,ab,kw (Word variations have been searched)  #5 MeSH descriptor: [Gastrointestinal Microbiome] explode all trees  #6 (Gastrointestinal Microbiome OR Gastrointestinal Microbiomes OR Microbiome, Gastrointestinal OR Gut Microbiome OR Gut Microbiomes OR Microbiome, Gut OR Gut Microflora OR Microflora, Gut OR Gut Microbiota OR Gut Microbiotas OR Microbiota, Gut OR Gastrointestinal Flora OR Flora, Gastrointestinal OR Gut Flora OR Flora, Gut OR Gastrointestinal Microbiota OR Gastrointestinal Microbiotas OR Microbiota, Gastrointestinal OR Gastrointestinal Microbial Community OR Gastrointestinal Microbial Communities OR Microbial Community, Gastrointestinal OR Gastrointestinal Microflora OR Microflora, Gastrointestinal OR Gastric Microbiome OR Gastric Microbiomes OR Microbiome, Gastric OR Intestinal Microbiome OR Intestinal Microbiomes OR Microbiome, Intestinal OR Intestinal Microbiota OR Intestinal Microbiotas OR Microbiota, Intestinal OR Intestinal Microflora OR Microflora, Intestinal OR Intestinal Flora OR Flora, Intestinal OR Enteric Bacteria OR Bacteria, Enteric):ti,ab,kw (Word variations have been searched)  #7 #1 or #2 or #3 or #4  #8 #5 or #6  #9 #7 and #8 |
| EMBASE | (Rhinitis, Allergic OR Rhinitis, Allergic, Seasonal OR Allergic Rhinitides OR Rhinitides, Allergic OR Allergic Rhinitis OR Seasonal Allergic Rhinitis OR Allergic Rhinitides, Seasonal OR Allergic Rhinitis, Seasonal OR Rhinitides, Seasonal Allergic OR Rhinitis, Seasonal Allergic OR Seasonal Allergic Rhinitides OR Pollen Allergy OR Allergies, Pollen OR Allergy, Pollen OR Pollen Allergies OR Pollinosis OR Pollinoses OR Hay Fever OR Fever, Hay OR Hayfever OR nasal allergy OR rhinallergosis) AND (Gastrointestinal Microbiome OR Gastrointestinal Microbiomes OR Microbiome, Gastrointestinal OR Gut Microbiome OR Gut Microbiomes OR Microbiome, Gut OR Gut Microflora OR Microflora, Gut OR Gut Microbiota OR Gut Microbiotas OR Microbiota, Gut OR Gastrointestinal Flora OR Flora, Gastrointestinal OR Gut Flora OR Flora, Gut OR Gastrointestinal Microbiota OR Gastrointestinal Microbiotas OR Microbiota, Gastrointestinal OR Gastrointestinal Microbial Community OR Gastrointestinal Microbial Communities OR Microbial Community, OR Gastrointestinal OR Gastrointestinal Microflora OR Microflora, Gastrointestinal OR Gastric Microbiome OR Gastric Microbiomes OR Microbiome, Gastric OR Intestinal Microbiome OR Intestinal Microbiomes OR Microbiome, Intestinal OR Intestinal Microbiota OR Intestinal Microbiotas OR Microbiota, Intestinal OR Intestinal Microflora OR Microflora, Intestinal OR Intestinal Flora OR Flora, Intestinal OR Enteric Bacteria OR Bacteria, Enteric) |
| CINAHL | TX (Rhinitis, Allergic OR Rhinitis, Allergic, Seasonal OR Allergic Rhinitides OR Rhinitides, Allergic OR Allergic Rhinitis OR Seasonal Allergic Rhinitis OR Allergic Rhinitides, Seasonal OR Allergic Rhinitis, Seasonal OR Rhinitides, Seasonal Allergic OR Rhinitis, Seasonal Allergic OR Seasonal Allergic Rhinitides OR Pollen Allergy OR Allergies, Pollen OR Allergy, Pollen OR Pollen Allergies OR Pollinosis OR Pollinoses OR Hay Fever OR Fever, Hay OR Hayfever OR nasal allergy OR rhinallergosis) AND TX (Gastrointestinal Microbiome OR Gastrointestinal Microbiomes OR Microbiome, Gastrointestinal OR Gut Microbiome OR Gut Microbiomes OR Microbiome, Gut OR Gut Microflora OR Microflora, Gut OR Gut Microbiota OR Gut Microbiotas OR Microbiota, Gut OR Gastrointestinal Flora OR Flora, Gastrointestinal OR Gut Flora OR Flora, Gut OR Gastrointestinal Microbiota OR Gastrointestinal Microbiotas OR Microbiota, Gastrointestinal OR Gastrointestinal Microbial Community OR Gastrointestinal Microbial Communities OR Microbial Community, OR Gastrointestinal OR Gastrointestinal Microflora OR Microflora, Gastrointestinal OR Gastric Microbiome OR Gastric Microbiomes OR Microbiome, Gastric OR Intestinal Microbiome OR Intestinal Microbiomes OR Microbiome, Intestinal OR Intestinal Microbiota OR Intestinal Microbiotas OR Microbiota, Intestinal OR Intestinal Microflora OR Microflora, Intestinal OR Intestinal Flora OR Flora, Intestinal OR Enteric Bacteria OR Bacteria, Enteric) |
| Web of science | (TS=(Rhinitis, Allergic OR Rhinitis, Allergic, Seasonal OR Allergic Rhinitides OR Rhinitides, Allergic OR Allergic Rhinitis OR Seasonal Allergic Rhinitis OR Allergic Rhinitides, Seasonal OR Allergic Rhinitis, Seasonal OR Rhinitides, Seasonal Allergic OR Rhinitis, Seasonal Allergic OR Seasonal Allergic Rhinitides OR Pollen Allergy OR Allergies, Pollen OR Allergy, Pollen OR Pollen Allergies OR Pollinosis OR Pollinoses OR Hay Fever OR Fever, Hay OR Hayfever OR nasal allergy OR rhinallergosis)) AND TS=(Gastrointestinal Microbiome OR Gastrointestinal Microbiomes OR Microbiome, Gastrointestinal OR Gut Microbiome OR Gut Microbiomes OR Microbiome, Gut OR Gut Microflora OR Microflora, Gut OR Gut Microbiota OR Gut Microbiotas OR Microbiota, Gut OR Gastrointestinal Flora OR Flora, Gastrointestinal OR Gut Flora OR Flora, Gut OR Gastrointestinal Microbiota OR Gastrointestinal Microbiotas OR Microbiota, Gastrointestinal OR Gastrointestinal Microbial Community OR Gastrointestinal Microbial Communities OR Microbial Community, OR Gastrointestinal OR Gastrointestinal Microflora OR Microflora, Gastrointestinal OR Gastric Microbiome OR Gastric Microbiomes OR Microbiome, Gastric OR Intestinal Microbiome OR Intestinal Microbiomes OR Microbiome, Intestinal OR Intestinal Microbiota OR Intestinal Microbiotas OR Microbiota, Intestinal OR Intestinal Microflora OR Microflora, Intestinal OR Intestinal Flora OR Flora, Intestinal OR Enteric Bacteria OR Bacteria, Enteric) |
