# Supplementary material for: Association between gut microbiota and allergic rhinitis: a systematic review and meta-analysis
Source: PeerJ. 2025 May 26;13:e19441. doi: 10.7717/peerj.19441 (PMC12121621; doi:10.7717/peerj.19441)
Supplement: Supplemental Information 2 [file peerj-13-19441-s002.docx]

Supplementary Table 2. Stool sample processing methods in the included studies

| **Study** | **Collection & handling by participant** | **Long-term storage** | **DNA extraction method** |
| --- | --- | --- | --- |
| Chiu, et al. 2019 | Fresh stools were collected in clean specimen bottles by parents from each child with instructions on proper method of collection | Samples were frozen immediately, and carefully transported to the laboratory where they were stored at −80°C until further use | Bacterial DNA was extracted from the same amount of feces (0.5 g) using a FastDNA Spin Kit for Feces (MP Biomedical, Solon, OH, USA) following the manufacturer's instructions. |
| Zhu, et al. 2020 | not mentioned | not mentioned | DNA was extracted from approximately 0.25 g of the fecal samples using the QIAamp PowerFecal DNA Kit (Qiagen, DE) according to the manufacturer’s instructions. |
| Liu, et al. 2020 | Samples were collected at home | Samples were mailed back to the hospital on the same day and frozen at −80℃ | The genome sequencing process and library construction were commissioned by Suzhou BiomeMatch Pharmaceutical Technology Co., LTD. |
| Watts, et al. 2021 | Subjects were provided with a sample collection kit and instructed to collect a stool sample within 24 h prior to their scheduled study visit. Collection instructions included not to contaminate the sample with urine or water and to store the sample at room temperature. | Stool samples were frozen at −80°C upon receipt until processing. | DNA was extracted from defrosted stool samples using the method, which included homogenisation, a combination of chemical and mechanical lysis (using silica/ zirconia beads; Daintree Scientific, St Helens, TAS, Australia), salt/ alcohol precipitation, and purification using a Qiagen DNAeasy kit (Qiagen, Hilden, Germany). |
| Zhou, et al. 2021 | not mentioned | Fecal samples from all participants were stored at − 80°C until analysis. | DNA was extracted from fecal samples using the E.Z.N.A.® soil DNA Kit, as described by the manufacturer (Omega BioTek, Norcross, GA, U. S.). |
| Sahoyama, et al. 2022 | Fresh faecal samples were collected in plastic containers containing glass beads (Tomy Seiko) and RNAlater Reagent (Life Technologies Japan). | The samples were transported at 4 °C to the laboratory. | The samples were transported at 4 °C to the laboratory. In the laboratory, the faecal samples (~0.2 g) were suspended in 15 mL phosphate-buffered saline (PBS) buffer and filtered with a 100-μm-mesh nylon filter (Corning) to remove human and eukaryotic cells and debris from the faecal sample. The debris on the filter was washed twice with PBS. The bacteria-enriched pellet was obtained by centrifugation of the filtrate at 9000×g for 10 min at 4 °C. The pellets were washed with 35 mL PBS once, further washed with TE20 buffer (10 mM Tris–HCl, 20 mM EDTA), and subjected to DNA extraction. |
| Lin, et al. 2022 | Samples were collected at home in both groups with MGI Easy Stool Sample Collection Kit. The collected samples were mailed back to the hospital on the same day. | Upon return to study staff, samples were stored at -80℃ until further processing. | Qualified genomic DNA samples were broken into approximately 400bp fragments by ultrasonic fragmentation CovarisM220 and library construction was performed with NEXTFLEX Rapid DNA-SeqKit. |
| Chiu, et al. 2023 | not mentioned | not mentioned | DNA was extracted using the FastDNA SPIN Kit for Feces (MP Biomedical) and fragmented using a Covaris M220 sonicator (Covaris, Inc.). |
| Zhang, et al. 2023 | The parents of the study participants were provided with a fecal sample collection kit and dry ice, and were instructed on how to collect the stool sample. The collection instructions emphasized the importance of avoiding contamination of the samples with urine or water, and the samples were frozen immediately, and laboratory using dry ice within 24h. | The samples were stored at a temperature of −80°C. | To analyze the diversity and composition of the microbial community, DNA was extracted from fecal samples, the concentration and purity were measured using the NanoDrop One (Thermo Fisher Scientific, MA, United States). |
| Wan, et al. 2023 | not mentioned | Researchers quickly freeze and transport stools samples on ice to laboratory −80°C freezers until further processing within 2 hours after sampling. | A frozen aliquot (200mg) of each fecal sample was processed using the Stool Genomic DNA Kit (CW2092S; CWBIO). |
