# Supplementary material for: Association between gut microbiota and allergic rhinitis: a systematic review and meta-analysis
Source: PeerJ. 2025 May 26;13:e19441. doi: 10.7717/peerj.19441 (PMC12121621; doi:10.7717/peerj.19441)
Supplement: Supplemental Information 3 [file peerj-13-19441-s003.docx]

Supplementary Table 3. Main results of alpha diversity

| Index | Sample sizes | | SMD | 95%CI | *I^2^* |
| --- | --- | --- | --- | --- | --- |
|  | AR | HC |  |  |  |
| Shannon index | 550 | 385 | -0.3938 | -0.9847; 0.1972 | 94% |
| Simpson index | 236 | 185 | -0.16 | -1.12; 0.80 | 96% |
| Chao1 index | 489 | 321 | 0.00 | -1.32; 1.32 | 97% |

AR: allergic rhinitis; HC: healthy control; SMD: standardized mean difference; CI:confidence intervals
