# Supplementary material for: Association between gut microbiota and allergic rhinitis: a systematic review and meta-analysis
Source: PeerJ. 2025 May 26;13:e19441. doi: 10.7717/peerj.19441 (PMC12121621; doi:10.7717/peerj.19441)
Supplement: Supplemental Information 4 [file peerj-13-19441-s004.docx]

Supplementary Table 4. Main results of relative gut microbiota abundance at phylum level

| Phylum | Sample sizes | | IQR | 95%CI | *I^2^* |
| --- | --- | --- | --- | --- | --- |
|  | AR | HC |  |  |  |
| Firmicutes | 75 | 75 | 0.05 | −0.10, 0.19 | 75% |
| Bacteroidetes | 144 | 122 | -0.12 | −0.42, 0.19 | 95% |
| Proteobacteria | 144 | 122 | -0.01 | −0.06, 0.03 | 92% |
| Actinobacteria | 144 | 122 | 0.00 | −0.09, 0.03 | 83% |

AR: allergic rhinitis; HC: healthy control; IQR: median interquartile range; CI:confidence intervals
