# Supplementary material for: Association between gut microbiota and allergic rhinitis: a systematic review and meta-analysis
Source: PeerJ. 2025 May 26;13:e19441. doi: 10.7717/peerj.19441 (PMC12121621; doi:10.7717/peerj.19441)
Supplement: Supplemental Information 5 [file peerj-13-19441-s005.docx]

Supplementary Table 5. Main results of subgroup analysis categorized by age

| Index | Age | Sample sizes | | SMD | 95%CI | *I^2^* |
| --- | --- | --- | --- | --- | --- | --- |
|  |  | AR | HC |  |  |  |
| Shannon index | Children | 163 | 136 | -0.16 | -0.54; 0.22 | 58% |
|  | Adults | 387 | 249 | -0.64 | -1.75; 0.48 | 97% |
| Chao1 index | Children | 120 | 89 | -0.06 | -0.34; 0.21 | 0% |
|  | Adults | 369 | 232 | 0.05 | -2.45; 2.54 | 97% |

AR: allergic rhinitis; HC: healthy control; SMD: standardized mean difference; CI:confidence intervals
