# Supplementary material for: Association between gut microbiota and allergic rhinitis: a systematic review and meta-analysis
Source: PeerJ. 2025 May 26;13:e19441. doi: 10.7717/peerj.19441 (PMC12121621; doi:10.7717/peerj.19441)
Supplement: Supplemental Information 6 [file peerj-13-19441-s006.docx]

Supplementary Table 6. Main results of subgroup analysis categorized by region

| Index | Region | Sample sizes | | SMD | 95%CI | *I^2^* |
| --- | --- | --- | --- | --- | --- | --- |
|  |  | AR | HC |  |  |  |
| Shannon index | China | 307 | 256 | -0.42 | -1.17; 0.32 | 95% |
|  | Australia | 57 | 23 | -0.51 | -1.00; 0.02 |  |
|  | Japan | 186 | 106 | -0.05 | -0.29; 0.19 |  |
| Chao1 index | China | 246 | 192 | 0.21 | -1.68; 2.09 | 98% |
|  | Australia | 57 | 23 | -0.86 | -1.36; 0.36 |  |
|  | Japan | 186 | 106 | -0.14 | -0.38; 0.10 |  |

AR: allergic rhinitis; HC: healthy control; SMD: standardized mean difference; CI:confidence intervals
