# Supplementary material for: Association between gut microbiota and allergic rhinitis: a systematic review and meta-analysis
Source: PeerJ. 2025 May 26;13:e19441. doi: 10.7717/peerj.19441 (PMC12121621; doi:10.7717/peerj.19441)
Supplement: Supplemental Information 7 [file peerj-13-19441-s007.docx]

Supplementary Table 7. Main results of subgroup analysis categorized by measurement method

| Index | Measurement method | Sample sizes | | SMD | 95%CI | *I^2^* |
| --- | --- | --- | --- | --- | --- | --- |
|  |  | AR | HC |  |  |  |
| Shannon index | 16S rRNA gene sequencing | 163 | 136 | -0.16 | -0.54; 0.22 | 58% |
|  | shotgun metagenomic sequencing | 369 | 232 | 0.05 | -2.45; 2.54 | 97% |

AR: allergic rhinitis; HC: healthy control; SMD: standardized mean difference; CI:confidence intervals
