# Supplementary material for: Association between gut microbiota and allergic rhinitis: a systematic review and meta-analysis
Source: PeerJ. 2025 May 26;13:e19441. doi: 10.7717/peerj.19441 (PMC12121621; doi:10.7717/peerj.19441)
Supplement: Supplemental Information 9 [file peerj-13-19441-s009.docx]

**Intended Audience for the Article**

This systematic review and meta-analysis is intended for the following audience:

1. **Clinicians (Allergy Specialists, General Practitioners, Pediatricians)**
   The findings of this study provide valuable insights for clinicians involved in the diagnosis and management of allergic rhinitis (AR). Although no significant relationship was found between gut microbiota diversity and AR, this analysis highlights the complexity of the relationship, suggesting that AR patients may not exhibit uniform gut flora imbalances. Clinicians may find this information useful for guiding treatment approaches, particularly in considering probiotic therapies for AR.
2. **Researchers**
   This meta-analysis offers a comprehensive evaluation of the existing literature on the association between gut microbiota and allergic rhinitis. Researchers studying the microbiome, immunology, or allergy-related conditions will find the synthesis of evidence, including statistical evaluations, important for identifying gaps in current knowledge and informing future research directions in the field of gut microbiota's role in allergic diseases.
3. **Policy Makers and Public Health Officials**
   Understanding the relationship between gut microbiota and allergic diseases, including AR, is of growing interest in public health. Although this study finds no significant evidence linking gut microbiota imbalances to AR, policymakers and public health officials may find the findings relevant when considering the broader implications of dietary, lifestyle, and environmental interventions aimed at managing or preventing allergic conditions.
4. **Healthcare Professionals (Pharmacists, Dietitians, and Other Allied Health Providers)**
   Given the potential use of probiotics and other gut-targeted therapies in treating allergic rhinitis, healthcare providers such as pharmacists and dietitians can benefit from understanding the current evidence on this topic. This article may help inform patient education, especially regarding the efficacy of probiotics and gut health in managing AR.
